# Knowledge and perception toward HIV pre-exposure prophylaxis among primary healthcare nurses in the Ingquza Hill Health Sub district, Eastern Cape, South Africa

**DOI:** 10.3389/fpubh.2026.1947859

**Published:** 2026-10-22

**Authors:** Ongama Mluleki Mtatambi, Bernard Hope Taderera

**Affiliations:** Department of Environmental Health, Faculty of Health Sciences, University of Johannesburg, Johannesburg, South Africa

**Keywords:** HIV infection, knowledge, perceptions, pre-exposure prophylaxis, primary healthcare nurses

## Abstract

**Background:**

Human immunodeficiency virus (HIV) is still a major public health concern in South Africa. Pre-exposure prophylaxis (PrEP) has been shown to effectively reduce the risk of new HIV infections. Despite this, research on healthcare workers' knowledge and perceptions of PrEP in rural OR Tambo districts remains limited.

**Aim:**

This study sought to assess the knowledge and explore the perceptions of HIV PrEP among primary healthcare (PHC) nurses in the Ingquza Hill Health Sub-District.

**Setting:**

The study was conducted at PHC facilities in the Ingquza Hill Health Sub-District, OR Tambo District, Eastern Cape Province, South Africa.

**Methods:**

A quantitative cross-sectional study was employed among primary healthcare nurses working at the Ingquza Hill Health Sub-District. Data were collected via a self-administered questionnaire. Data were analyzed using descriptive statistics, chi-square tests and binary logistic regression. Statistical significance was set at *p* < 0.05.

**Results:**

Among the 157 nurses surveyed, most showed a strong understanding of PrEP. Many could identify its definition (87.3%), eligibility criteria (86.6%), and preferred regimen (87.9%). In contrast, fewer nurses were aware of follow-up protocols (53.5%) and risk compensation (46.6%). The results also showed that knowledge of counseling messages, contraindications, and when to stop PrEP varied by professional category (*p* < 0.05).

**Conclusion:**

Primary Healthcare nurses in the Ingquza Hill Health Sub-District understood PrEP well and held mostly positive views of it. Nevertheless, some had trouble complying with protocols and did not fully understand risk compensation. The data show that more training for nurses could make PrEP work better in rural areas where HIV rates are high.

## 1 Introduction

Human Immunodeficiency Virus (HIV) is still a major global health problem and has caused about 44.1 million deaths worldwide (1). It impacts people in every region. By the end of 2024, about 40.8 million people were living with HIV, with 65% of them in Africa (2). In 2024, around 630,000 people died from HIV-related causes, and about 1.3 million people became newly infected (1). In 2025, South Africa had an estimated 8.15 million people living with HIV, which is about 12.9% of its population (2). Even with advances in treatment and prevention, the region still sees high numbers of new HIV cases (3). In 2023, South Africa reported about 149,000 new cases, with 91,000 among women and 50,000 among men (4). These numbers show that new HIV infections are still much higher than zero, which is the United Nations Sustainable Development Goals (SDGs) target to reduce new infections by 95% by 2030 (5). The South African government is committed to ensuring that, by 2030, 95% of people living with HIV know their status, 95% of those diagnosed receive antiretroviral therapy (ART), and 95% of those on treatment achieve viral suppression (6).

Pre-exposure prophylaxis (PrEP) is the use of antiretroviral medication by HIV-negative individuals to reduce their risk of HIV acquisition (7). PrEP has been shown to be highly effective when taken consistently and is recognized as an essential component of comprehensive HIV prevention programmes (8). South Africa has incorporated PrEP into national HIV prevention guidelines and progressively expanded access among populations at substantial risk of HIV infection (9). PrEP may be taken continuously or during periods of increased risk (9). Daily oral PrEP has been shown to reduce the risk of HIV acquisition by up to 92% (10). Although progress has been made, HIV continues to pose a major global public health threat (11). While recent progress has been achieved, South Africa still reports a substantial number of new HIV infections annually, highlighting the urgent need to strengthen prevention strategies (9). Stronger efforts will help meet national and global goals to lower new HIV infections and move closer to achieving Sustainable Development Goal 3 by 2030 (5).

The widespread expansion and acceptance of PrEP depend on healthcare professionals having thorough knowledge of PrEP, communicating clearly with patients, and prescribing it confidently (12, 13). Primary healthcare nurses, who often encounter HIV-negative individuals at risk of infection, play a vital role in identifying patients who are eligible for PrEP. Limited PrEP uptake and awareness present opportunities to reach at-risk populations (14). These data point out the need to assess nurses' knowledge and attitudes worldwide, as their expertise directly affects patient care and HIV prevention.

Studies from around the world show that nurses usually see PrEP as an effective way to prevent HIV, but there are still significant gaps in their knowledge. In high-income countries, there is general awareness of PrEP (15). However, many healthcare providers lack confidence in eligibility criteria, patient monitoring, and side-effect management, resulting in low prescription rates (5, 16). Previous studies conducted in South Africa and other African countries have reported varying levels of knowledge and differing perceptions regarding PrEP among healthcare workers (17). Knowledge gaps, misconceptions and concerns regarding behavioral risk compensation have been identified as important barriers to PrEP implementation (18, 19). However, limited evidence exists regarding the knowledge and perceptions of nurses working in rural primary healthcare settings in the Eastern Cape Province. This study therefore aimed to assess the knowledge and perceptions of HIV pre-exposure prophylaxis among primary healthcare nurses in the Ingquza Hill Health Sub-District of the OR Tambo District Municipality.

## 2 Methodology

### 2.1 Study design and setting

A quantitative, cross-sectional survey design was utilized to collect primary data regarding PrEP knowledge and perceptions among primary healthcare nurses. This approach was suitable for assessing the current state of knowledge and perceptions at a specific point in time. The research was conducted in the Ingquza Hill Health Sub-District of the OR Tambo District Municipality, Eastern Cape Province, South Africa. The healthcare system in this district faces considerable strain due to poor infrastructure, limited resources, and a high disease burden (20). Primary healthcare nurses in this district face high caseloads, staff shortages, limited training, poor working conditions, and challenging social and economic issues. These issues make it hard to implement and promote PrEP (21, 22). As a result, knowledge gaps and misconceptions persist, notably regarding eligibility, follow-up, and risk compensation (23–25).

### 2.2 Participants and sampling

The study used a single-stage cluster sampling design to recruit participants within the resource-limited OR Tambo District Municipality. The district comprises five geographical health sub-districts, which served as the primary sampling clusters. Due to administrative and funding constraints, Ingquza Hill Health Sub-District was purposively selected as the target cluster because it represented a rural, high-prevalence setting. A census approach was then applied within the selected cluster to minimize intra-cluster bias and maximize representation. All 21 primary healthcare facilities in the Ingquza Hill Health Sub-District were included, and all primary healthcare nurses meeting the inclusion criteria at these facilities were invited to participate (26).

The sample size was calculated using Epi Info version 7.2. The initial sample size was set at 135. To account for possible dropouts and non-responses, a 25% contingency was added (135 x 0.25 = 33.75). Therefore, the adjusted target sample size was 169 primary healthcare nurses (135 + 34 = 169) (27). The response rate was sufficient to ensure statistical power and broad representativeness. The inclusion criteria required that participants be registered with the South African Nursing Council and employed full-time within the Ingquza Hill Health Sub-District.

### 2.3 Data collection instruments and procedures

Data were collected using a structured, self-administered questionnaire with closed-ended questions. The questionnaire was adapted from a validated HIV prevention study conducted in the Ekurhuleni district and reviewed by nurse instructors to make sure it was relevant to pre-exposure prophylaxis (PrEP) (7). The questionnaire comprised three sections:

- Section A: Demographic characteristics, assessed gender, age, professional category, years of experience, and highest qualifications.
- Section B: PrEP knowledge items, questions assessed understanding of PrEP definitions, eligibility criteria, contraindications, follow-up monitoring, counseling messages, discontinuation criteria, and treatment regimens.
- Section C: PrEP perception items assessed attitudes toward PrEP implementation, risk compensation, ethical concerns, workload implications, and willingness to recommend PrEP

A pilot study was further done with 10 nurses testing the questionnaire for clarity, feasibility, and relevance. Minor changes were made based on their feedback. Cronbach's alpha values was 0.72 to 0.75 for both the knowledge and perception sections, which shows acceptable reliability. Data collection took place from January to February 2026. Before participating, everyone was informed about the study's goals, confidentiality, and the voluntary nature of participation. All participants gave written informed consent.

### 2.4 Data analysis

Data were analyzed using IBM SPSS Statistics version 30. Descriptive statistics, including frequencies, percentages, means and standard deviations, were used to summarize study variables. For the knowledge assessment, each correct response was assigned a score of one and each incorrect response a score of zero. Individual scores were summed across all knowledge items and converted to percentages. The overall knowledge score reported in this study represents the mean percentage score across all participants (23).

Associations between categorical variables were assessed using chi-square tests or Fisher's exact tests where chi-square assumptions were not met. Statistical significance was set at p < 0.05. Associations between professional category and selected knowledge and perception variables were further explored using binary logistic regression analysis. The dependent variable was professional category, coded as Professional Nurse (0) and Enrolled Nurse (1). Individual knowledge and perception items were entered separately as independent variables to examine differences between two nursing categories. Odds ratios (ORs) with 95% confidence intervals (95% CI) were calculated. Statistical significance was assessed at *p* < 0.05 (23).

### 2.5 Ethical approval

Ethical approval was granted by the University of Johannesburg Faculty of Health Sciences Higher Education Committee (Reference: HDC-01-91-2025), the Research Ethics Committee (Reference: REC-3929-2025), and the Eastern Cape Department of Health. Additional permissions were obtained from the OR Tambo District Manager and the Ingquza Hill Health Sub-District Manager. All participants provided written informed consent, and participation was voluntary. Confidentiality and anonymity were maintained throughout the study.

## 3 Results

### 3.1 Knowledge of PrEP among PHC nurses

A total of 157 nurses participated in the study, making 92.9% (157/169 x 100 = 93%) of the adjusted target of 169 and exceeding the minimum requirement of 135. Table 1 shows that a majority (87.3%, 137/157) correctly identified PrEP as an antiretroviral (ARV) drug taken by HIV-negative people before possible exposure. HIV-negative rapid antibody testing was recognized by 86.6% of respondents as a requirement for PrEP eligibility. Similarly, 81.5% (128/157) correctly identified candidates for PrEP. Moreover, 78.3% selected the comprehensive “all of the above” option, indicating a broad understanding of the counseling themes, as shown in Table 1.

**Table 1:** Distribution of PrEP knowledge among respondents.

Characteristic	Response	Frequency (*n* = 157)	Proportion %
PrEP definition	Lifelong treatment with ARV drugs	13	8.3
	ARV medication taken within 72 h after exposure to HIV	7	4.5
	ARV medication taken by an HIV-negative person before exposure to HIV	137	87.3
Eligibility criteria for PrEP	HIV-negative rapid antibody test	136	86.6
	Negative tuberculosis screening	1	0.6
	None of the above	20	12.7
PrEP screening questions	Have you had sex without a condom?	21	13.4
	Have you had sex under the influence of alcohol or drugs?	0	0
	Have you ever had sexually transmitted infections?	0	0
	All of the above are correct	136	86.6
Candidates for PrEP	People who request PrEP	24	15.3
	Men who have sex with men	2	1.3
	People with HIV infection	3	1.9
	A and B	128	81.5
Counseling messages before PrEP initiation	Definition of PrEP	5	3.2
	How to start and stop PrEP	25	15.9
	Follow-up visits schedule	4	2.5
	All of the above are correct	123	78.3
Contraindications for PrEP	Being on lifelong ART	136	86.6
	Being diagnosed with an STI	21	13.4
PrEP follow-up visit Reasons for discontinuing PrEP	Address side effects	156	99.4
	Education about the dangers of smoking	1	0.6
	Testing HIV-positive infection	41	26.1
	Developing renal impairment CrCl < 60Ml/min	5	3.2
	All of the above	111	70.7
Preferred PrEP regimen in South Africa	Tenofovir, Emtricitabine, and Efavirenz	3	1.9
	Dolutegravir	16	10.2
	Tenofovir and Emtricitabine	138	87.9
Is PrEP intended for both treatment and prevention?	True	45	28.6
	False	112	71.3
Schedule for PrEP doses	Once daily	149	94.9
	Twice daily	8	5.1
Are patient follow-up visits on PrEP done monthly	True	73	46.5
	False	84	53.5

Table 1 further illustrates that 86.6% (135/157) of participants correctly identified contraindications. Similarly, the vast majority (99.3%; 156/157) indicated the need to discuss adverse effects at follow-up appointments. Respondents 46.5% (73/157) incorrectly mentioned that patients should have monthly follow-up appointments. Most respondents 71.3% (112/157) correctly disagreed with the statement that PrEP is intended for both treatment and prevention. Majority of respondents 87.9% (138/157) correctly selected Tenofovir and Emtricitabine as South Africa's preferred treatment regimen. Respondents 94.9% (148/157) knew that PrEP is taken once daily. There was confusion regarding the frequency of follow-up visits to monitor side effects and toxicities. The overall knowledge score reported in this study was 82 %, calculated as the arithmetic mean of the 12 correct response dimensions, indicating generally good PrEP knowledge among participating primary healthcare nurses in the Ingquza Hill Health Sub-District as displayed in Table 2.

**Table 2:** Distribution of correct responses on PrEP knowledge items.

Knowledge item	Correct Response	% Percentage
Definition of PrEP	ARV medication taken by HIV-negative persons before exposure	87.3 %
Eligibility criteria	HIV-negative rapid antibody test	86.6%
Screening questions	All the above are correct	86.6%
Potential candidates	People who request PrEP + MSM	81.5%
Counseling message before initiation	All the above are correct	78.3 %
Contraindications	Being on lifelong ART	86.6%
Follow-up visit content	Address side effects	99.4%
Reasons for discontinuation	All of the above (HIV-positive + renal disease)	70.7%
Preferred regimen in South Africa	Tenofovir + Emtricitabine	87.9%
Dosage frequency	Once daily	94.9%
PrEP is intended for treatment and prevention	False	71.3%
Patient follow-up visit on PrEP	False	53.5%
Average knowledge level		82%

### 3.2 Perceptions of PrEP among PHC nurses

As shown in Table 3, most respondents had positive perceptions of PrEP. The great majority felt that starting PrEP is a good idea 89.8% (141/157), that teaching people about PrEP is very important 97.5% (153/157). Some concerns remained. About 52.2% (82/157) thought PrEP made people less likely to use condoms. Most participants 75.2% (118/157) felt PrEP education is crucial, and 75.2% (118/157) perceived that PrEP empowers women who are unable to negotiate condom use. Most respondents 77.7% (122/157) disagreed that PrEP exerts undue pressure on healthcare workers, while 19.7% (31/157) agreed. Approximately 67.5% (106/157) of respondents did not view the dosing schedule as a barrier, while 13.4% (21/157) considered it a barrier. Only 5.7% (9/157) agreed that it is ethically wrong to prescribe PrEP to healthy patients.

**Table 3:** Distribution of PrEP perceptions among respondents.

Characteristic	Response	Frequency (*n* = 157)	Proportion %
PrEP roll-out	Agree	141	89.8
	Disagree	12	7.6
	Not sure	4	2.5
PrEP overburdens staff	Agree	31	19.7
	Disagree	122	77.7
	Not sure	4	2.5
PrEP reduces condom use	Agree	82	52.2
	Disagree	63	40.1
	Not sure	12	7.6
PrEP promotes risky sexual behavior	Agree	74	47.1
	Disagree	68	43.3
	Not sure	15	9.6
Behavioral interventions work better than PrEP	Agree	35	22.3
	Disagree	94	59.9
	Not sure	28	17.8
The dosage schedule may act as a barrier to PrEP usage	Agree	21	13.4
	Disagree	106	67.5
	Not sure	30	19.1
PrEP education is an essential part of HIV prevention	Agree	153	97.5
	Disagree	4	2.5
	Not sure	0	0
PrEP empowers women who are unable to negotiate condom use	Agree	118	75.2
	Disagree	26	16.6
	Not sure	13	8.3
It is unethical to prescribe PrEP to healthy individuals	Agree	9	5.7
	Disagree	143	91.1
	Not sure	5	3.2
I am always willing to recommend PrEP to potential users	Agree	147	93.6
	Disagree	8	5.1
	Not sure	2	1.3

### 3.3 Inferential statistics

In the chi-square analysis, professional responsibilities were grouped into two categories to explore their associations. Operational managers were combined with professional nurses, while enrolled nurses and enrolled nursing assistants were grouped as enrolled nurses. The relationship between professional category and knowledge and attitudes toward PrEP was assessed using chi-square analysis. Several associations were statistically significant (p < 0.05), indicating that professional nurses and enrolled nurses differed in their responses.

#### 3.3.1 Association between professional category and knowledge of PrEP

PrEP knowledge was compared between professional nurses (*n* = 115) and enrolled nurses (*n* = 42) using chi-square testing. There was no significant association (χ^2^ (2) = 0.135, *p* = 0.935). In both groups, PrEP was appropriately identified as an antiretroviral drug used by HIV-negative persons before exposure. In addition to the PrEP knowledge variable, the eligibility criterion variable did not demonstrate significant variation across groups (χ^2^ (2) = 0.929, *p* = 0.629). Both groups identified HIV-negative antibody testing as the key criterion.

No significant difference emerged between professional nurses and enrolled nurses on screening questions (χ^2^ (1) = 0.536, *p* = 0.464), and both groups demonstrated adequate understanding of screening requirements. However, as shown in Table 4, highly significant differences appeared across more complex guideline areas selected to assess higher-level guideline understanding. Professional nurses were significantly more likely to identify comprehensive, multi-component initiation counseling packages (79.7% vs. 20.3%; χ^2^ (3) = 13.553, *p* = 0.003). Similarly, professional nurses demonstrated significantly greater accuracy in identifying active lifelong ART as an absolute contraindication, whereas enrolled nurses frequently misidentified an acute STI diagnosis as an exclusion criterion (79.4% vs. 66.7%; χ^2^ (1) = 19.711, *p* < 0.001).

**Table 4:** Association between professional category and knowledge of PrEP.

Knowledge item	*χ^2^* value	*df*	*p*-value	Significance
Counseling messages before PrEP initiation	13.55	3	0.003	Significant
Contraindications for PrEP	19.71	1	0.001	Significant
Reasons for discontinuing PrEP	7.04	2	0.030	Significant
PrEP intended for treatment and prevention	15.78	1	0.001	Significant
Monthly follow-up for side effects and toxicities	11.72	1	0.001	Significant
Definition of PrEP	0.14	2	0.935	Non-significant
Eligibility criteria for PrEP	0.93	2	0.629	Non-significant
Screening questions for PrEP	0.54	1	0.464	Non-significant
Potential candidates for PrEP	3.26	3	0.353	Non-significant
Preferred PrEP regimen	2.74	2	0.254	Non-significant
Prescribed once-daily dosing frequency	0.01	1	0.909	Non-significant

There were also significant differences in reasons for discontinuation (χ^2^ (2) = 7.037, *p* = 0.030), as shown in Table 4. Professional nurses were more likely to select “all of the above” as the correct answer (79.3%), whereas enrolled nurses tended to select individual reasons such as “testing HIV-positive.” There was also a significant difference in understanding of PrEP's purpose (χ^2^ (1) = 15.776, *p* < 0.001). Enrolled nurses (48.9%) incorrectly believed PrEP was for both treatment and prevention, while 51.1% of professional nurses responded correctly. Responses regarding monthly follow-up differed significantly (χ^2^ (1) = 11.721, *p* < 0.001), as displayed in Table 4.

#### 3.3.2 Association between professional category and perceptions of PrEP

Chi-square tests were also used to examine differences between the two categories regarding PrEP perceptions. The association between professional category and perceptions of PrEP is presented in Table 5 below. The first item, indicating the “PrEP rollout decision”, did not differ significantly (χ^2^ (2) = 0.688, *p* = 0.709). Both categories agreed that the rollout is a good decision. “PrEP reduces condom use” showed significant differences 69.5% vs. 30.5% (χ^2^ (2) = 6.670, *p* = 0.036). A significant difference was also found regarding the dosing schedule as a barrier to PrEP (χ^2^ (2) = 10.313, *p* = 0.006), as displayed in Table 5. Uncertainty was reported by 35.7% of enrolled nurses and 64.3% of professional nurses.

**Table 5:** Association between Professional Category and Perceptions of PrEP.

Perception item	*χ^2^* value	*Df*	*p*-value	Significance
PrEP reduces condom use	6.67	2	0.036	Significant
Ethical concerns about prescribing PrEP	9.33	2	0.009	Significant
Dosage schedule as a barrier to PrEP usage	10.31	2	0.006	Significant
PrEP roll-out is a good decision	0.69	2	0.709	Non-significant
PrEP overburdens staff	1.50	2	0.472	Non-significant
PrEP promotes risky sexual behavior	2.33	2	0.312	Non-significant
Behavioral interventions work better than PrEP	1.49	2	0.475	Non-significant
PrEP education is essential	0.01	1	0.936	Non-significant
PrEP empowers women	2.48	2	0.290	Non-significant
Willingness to recommend PrEP	5.55	2	0.062	Non-significant

Responses to the statement “It is unethical to prescribe PrEP” indicated a significant difference (χ^2^ (2) = 9.325, *p* =0.009). More enrolled nurses than professional nurses agreed that providing PrEP to healthy adults is unethical (66.7% compared to 33.3%). Other factors, including workload, risky sexual behavior, women's empowerment, and willingness to recommend PrEP, did not differ significantly between the groups. Nevertheless, the likelihood of recommending PrEP approached statistical significance (χ^2^ (2) = 5.550, *p* = 0.062), as shown in Table 5.

#### 3.3.3 Association of education level and PrEP knowledge

Academic qualifications showed no significant relationship with basic baseline definitions χ^2^ (6, *N* = 157) = 2.39, *p* = 0.88. Similarly, eligibility criteria for initiation of PrEP did not show a strong association with education, χ^2^ (6, *N* = 157) = 2.37, *p* = 0.88. The association between education level and knowledge of counseling messages on initiation of PrEP approached statistical significance. However, it did not reach the conventional significance level of 0.05, χ^2^ (9, *N* = 157) = 16.76, *p* = 0.053. Education level was also strongly associated with knowledge of PrEP contraindications, with a negative correlation (*r* = 0.30, *p* < 0.001). Education was significantly associated with understanding that PrEP is intended to treat and prevent HIV (χ^2^ (6, *N* = 157) = 17.15, *p* = 0.009). It was also significantly related to knowledge of appropriate follow-up schedules, χ^2^ (3, *N* = 157) = 11.87, *p* = 0.008.

#### 3.3.4 Association of education level and PrEP perception

Education was significantly associated with the perception that pre-exposure prophylaxis (PrEP) promotes risky behavior, χ^2^ (6, *N* = 157) = 8.71, *p* = 0.011. Additionally, the perception that dosage is a barrier to PrEP use was also significant, χ^2^ (6, *N* = 157) = 14.46, *p* = 0.025. Furthermore, perceptions of ethics, such as whether it is unethical to offer PrEP to healthy people, were significantly associated with education, χ^2^ = 18.06, *p* = 0.006.

### 3.4 Logistic regression analysis

Binary logistic regression analyses were performed to assess the associations between professional category and selected variables related to PrEP knowledge and perceptions. The dependent variable was professional category, defined as professional nurse vs. enrolled nurse. A significant association was identified for knowledge of PrEP contraindications (OR = 7.71; 95% CI: 2.84-20.93; *p* < 0.001), demonstrating that responses varied significantly according to professional category. Significant associations were also observed for knowledge of PrEP discontinuation (OR = 0.37; 95% CI: 0.17-0.80; *p* = 0.011) and monthly follow-up monitoring (OR = 0.28; 95% CI: 0.13-0.59; *p* = 0.001). Among perception variables, significant associations were observed for perceptions that PrEP reduces condom use (*p* = 0.038), dosage schedules as barriers to PrEP use (*p* = 0.009), and the ethics of prescribing PrEP to healthy individuals (*p* = 0.009). Most other knowledge and perception variables were not significantly associated with professional category. The logistic regression results are presented in Table 6.

**Table 6:** Logistic regression results for selected knowledge and perception variables.

Characteristics	OR	95% CI	*p*-value
Contraindications for PrEP	7.71	2.84–20.93	< 0.001
Reasons for discontinuing PrEP	0.37	0.17–0.80	0.011
Monthly follow-up monitoring	0.28	0.13–0.59	0.001
PrEP intended for treatment and prevention of HIV	0.23	0.11–0.49	< 0.001
PrEP reduces condom use	0.48	0.22–1.08	0.075
Dosage schedule as barrier to PrEP use	3.20	0.93–10.98	0.064
Prescribing PrEP to healthy individuals is unethical	0.17	0.04–0.71	0.015

## 5 Discussion

### 5.1 Knowledge of PrEP among PHC nurses

Effective scaling up of HIV prevention depends on having primary healthcare nurses who are well informed about clinical eligibility and the procedures for implementation. The data from this study show that the primary healthcare nurses in the Ingquza Hill Health Sub-District have a good initial understanding of the key PrEP concepts, since 81% (127/157) obtained an acceptable overall score for their knowledge. This baseline knowledge aligns with findings from similar studies in Tshwane and Ekurhuleni, where clinical groups showed broad awareness of how PrEP works. (7, 23).

Although the evidence indicates a basic level of proficiency, major difficulties remain in operational understanding. Among primary healthcare nurses in the Ingquza Hill Health Sub-District, 87.3% % (137/157) gave the correct definition of PrEP. Yet a number of the participants confused PrEP with post-exposure prophylaxis (PEP) or with lifelong antiretroviral therapy. This confusion reflects deficiencies in clinical training reported in sub-Saharan Africa, where concurrent antiretroviral protocols frequently lead providers to misunderstand the topic (28).

A major finding of this study is the severe deficit regarding longitudinal monitoring and follow-up timelines. Around 46.5% (73/157) of respondents incorrectly identified the required monthly patient follow-up intervals. This gap exposes a critical programmatic vulnerability. Safe PrEP maintenance requires systematic clinical monitoring to screen for acute HIV seroconversion, track medication adherence, and actively manage potential drug-induced toxicities (9). This is similar to findings from Ethiopia and Ghana, where nurses were unsure about monitoring and eligibility for PrEP initiation (3, 17). Studies conducted in the United States and Europe indicate that health care providers commonly lack knowledge, notably about adherence counseling and monitoring requirements (15). Collectively, these data show the requirement for consistent training, concise guidelines, and ongoing professional development to enable nurses worldwide to provide safe and effective PrEP services.

Routine monitoring is important for assessing adherence to PrEP, finding potential side effects, and determining whether PrEP is still needed (9). The OR Tambo District study found major gaps, indicating that planned PrEP training and its inclusion in nursing education are urgently needed, in line with South Africa's 2021 PrEP guidelines (7, 9). To end the AIDS epidemic and other communicable diseases by 2030, it is necessary to expand focused prevention strategies, cut new infections by 90%, and lower AIDS-related deaths (10). Building expertise is also key to achieving the UNAIDS 95-95-95 targets and Sustainable Development Goal 3, both of which aim to end the HIV epidemic by 2030 (5, 6).

Furthermore, our inferential analysis revealed that clinical knowledge varies significantly by professional nursing category (*p* < 0.05). Professional nurses demonstrated a comprehensive understanding of counseling requirements, reasons for clinical discontinuation, and formal contraindications. In sharp contrast, enrolled nurses were significantly more likely to select incomplete counseling packages or incorrectly identify active sexually transmitted infections (STIs) as an absolute contraindication to PrEP initiation. Under national guidelines, an active STI is an explicit indicator of elevated risk that requires immediate PrEP initiation rather than exclusion (9). Because nurses routinely manage frontline triaging in rural, resource-limited clinics, these specific knowledge gaps highlight the urgent need for targeted, tier-specific clinical mentorship and continuing education programs (22).

### 5.2 Perceptions of PrEP among PHC nurses

Frontline acceptance is essential to a successful provider-initiated prevention strategy (23). Nurses in the Ingquza Hill Health sub-district showed overwhelmingly positive attitudes toward the PrEP rollout, with 89.8% (141/157) endorsing the current expansion and 93.6% (147/157) stating they were willing to recommend PrEP to eligible clients. Additionally, 75.2% (118/157) of respondents recognized PrEP as an important mechanism to empower women and adolescent girls who face structural barriers to condom negotiation. This finding directly supports the gender-responsive public health targets outlined in Sustainable Development Goal 5 (5, 7).

Environmental and work-related context formed these attitudinal trends. Primary healthcare nurses in the Ingquza Hill Health Sub-District function within a strained rural public health ecosystem characterized by infrastructure deficits, high daily patient volumes, and severe staffing shortages (22). In spite of these difficult conditions, 77.7% (122/157) of surveyed nurses explicitly disagreed that PrEP expansion overburdens daily clinic operations. This suggests a strong devotion to preventive health. This aligns with the World Health Organization's plan to expand PrEP access worldwide and supports South Africa's goal of expanding nurse-led PrEP programmes (23). Previous studies in South Africa found more uncertainty and doubt about PrEP. However, recent results show that acceptance has increased and skepticism has decreased (7, 29). Research from 2022 to 2023 also shows that starting PrEP with nurses in primary healthcare clinics is effective and widely accepted. Nurses are key in counseling patients and supporting them to stay on their treatment (30, 31).

However, positive attitudes toward the system are tempered by ongoing worries about behavioral risk compensation, as shown by primary healthcare nurses in Ingquza Hill Health Sub-District selected to illustrate this point. Among these nurses, 52.2% (82/157) believe that PrEP leads to reduced consistent condom use, and 47.1% (74/157) claim that it directly promotes riskier sexual behavior among young people in the area. These concerns about behavioral disinhibition correspond to global clinical trends, even though ample epidemiological evidence shows that structured PrEP delivery, combined with risk-reduction counseling, does not increase unsafe sexual behavior (8).

Professional status and education level influenced perceptions of these issues. Enrolled nurses reported significantly greater ethical discomfort regarding the prescription of antiretroviral drugs to healthy, HIV-negative individuals (*p* = 0.009). They were also more likely to perceive daily dosing schedules as a long-term barrier that patients would be unable to overcome (*p* = 0.006).

These data suggest that nurses in lower professional positions may view preventive medicine primarily as a means to cure, rather than to prevent, illness. This stresses the need for training programs to go beyond technical guidelines (17, 32). Educational efforts should incorporate real-world behavioral data and moral guidelines that demonstrate the wider community benefits of biomedical prevention. Dealing with these misconceptions among providers is essential to improving care and preventing inappropriate denial of treatment (13, 16).

### 5.3 Implications for practice and policy

The results show the need for systematic training courses to help nurses better understand who is eligible for PrEP, how to manage follow-up, and how to provide counseling. It is also important to tackle misunderstandings about risk compensation and condom use, since almost half of the respondents thought that PrEP could lead to riskier sexual behavior. The Department of Health should provide regular nurse training and strengthen community awareness campaigns. Policymakers also need to create supportive policies that will improve access to PrEP (32).

To successfully integrate PrEP into routine primary healthcare services, clear protocols, strong mentorship, and regular supervision must be combined to ensure high-quality PrEP delivery (22). Standardized training and ongoing professional development equip nurses with the skills they need to deliver PrEP effectively, thereby directly supporting South Africa's 95-95-95 HIV targets (5, 33). This strategy also corresponds to the World Health Organization's universal health coverage goals, which aim to prepare frontline providers to offer key prevention services and connect global priorities with local outcomes (17, 34).

### 5.4 Limitations of the study

This study took place in one of the five OR Tambo sub-districts due to budget and administrative limitations, so the findings do not extend to the whole district or province. In addition, using both interviews and focus groups would help lower reliance on self-reported data and afford a deeper look into nurses' experiences with PrEP training and implementation. In spite of these limitations, the study still gives important knowledge of PrEP knowledge and perceptions among PHC nurses in a high-prevalence, resource-limited setting. Consequently, future research into PrEP may need dedicated grant funding to achieve wider geographic coverage and more rigorous study designs, producing stronger evidence to inform public health policies on HIV prevention and control and to help advance both the Sustainable Development Goal 3 and Universal Health Coverage.

## 6 Conclusions

Primary healthcare nurses in the communities such as Ingquza Hill Health Sub-District have a solid grasp of the main mechanisms of PrEP and show a strong commitment to integrating it into clinical settings in rural areas. It is concluded that most primary healthcare nurses correctly identify the eligibility criteria for PrEP, are knowledgeable about the recommended national drug regimens, and view PrEP as an intervention that enables people to take greater control of their health. This has positive consequences for expanding access to HIV prevention services, reducing new HIV infections, and strengthening progress toward ending the HIV/AIDS epidemic. Moreover, clinical competence varies considerably by professional level, with enrolled nurses showing much higher rates of confusion about the guidelines and greater ethical hesitation than professional nurses.

To close these gaps in the rural communities of the Eastern Cape, the Eastern Cape Department of Health needs to deploy targeted, tier-specific clinical mentorship and continuing nursing education. Rectifying these basic misconceptions is essential to optimize provider-initiated delivery, support South Africa's national HIV prevention targets, and advance toward the Sustainable Development Goal 3 of universal health coverage.

## Data Availability

The raw data supporting the conclusions of this article will be made available by the authors, without undue reservation.

## References

[1] World Health Organization. HIV and AIDS (2025). Available online at: https://www.who.int/news-room/fact-sheet/detail/hiv-aids (Accessed February 28, 2026).

[2] Statistics South Africa. Mid-Year Population Estimates (2025). Available online at: https://www.statssa.gov.za/?p=18613 (Accessed April 10, 2026).

[3] Asabor EN Lett E Moll A Shenoi SV. “We've got our own beliefs, attitudes, myths”: a mixed methods assessment of rural South African health care workers' knowledge of and attitudes towards PrEP implementation. AIDS Behav. (2021) 25:2517–32. doi: 10.1007/s10461-021-03213-z PMC8450017 33763802

[4] Low M. One in Four People with HIV Not on Treatment, According to New Estimates (2024). Available online at: https://www.spotlightnsp.co.za/2024/04/04/one-in-four-people-with-hiv-not-on-treatment-according-to-new-estimates/ (Accessed May 10, 2026).

[5] UNAIDS. Global HIV Prevention Progress Report. Geneva: UNAIDS (2025).

[6] Madela EIY Gregson CL Paruk F Burton AJ Patel R Wilson H . UNAIDS 95-95-95 targets in older people living with HIV in urban and rural KwaZulu-Natal, South Africa. BMC Infect Dis. (2026) 26:972. doi: 10.1186/s12879-026-13273-y PMC13188679 41947066

[7] Mahlare E Ramutshila E Musekiwa A Kuonza L Mabuto T. Knowledge, attitudes and practices of oral HIV pre-exposure prophylaxis (PrEP) among healthcare workers in the Ekurhuleni District, South Africa. South Afr MJ. (2023) 113:46–6. doi: 10.7196/SAMJ.2023.v113i12.1171 38525627

[8] World Health Organization. Differentiated and Simplified Pre-Exposure Prophylaxis for HIV Prevention. Update to WHO Implementation Guidance: Technical Brief. Geneva: World Health Organization (2022).

[9] South Africa National Department of Health. Updated Guidelines for the Provision of Oral Pre-Exposure Prophylaxis (PrEP) to Persons at Substantial Risk of HIV Infection. Pretoria: Department of Health (2021).

[10] Sundareshan V Mangat R Koirala J. Pre-exposure Prophylaxis for HIV Prevention. PubMed. Treasure Island, FL: StatPearls Publishing (2022). Available online at: https://www.ncbi.nlm.nih.gov/books/NBK507789/ (Accessed October 13, 2025).

[11] Granich R Gupta S Williams B. Ending AIDS: Progress towards the 95-95-95 targets. Lancet HIV. (2022) 9:e730–8. doi: 10.1097/COH.0000000000000761

[12] Gant JE. Knowledge, Experiences, and Perceptions of Nurse Practitioners in Mississippi Regarding Pre-exposure Prophylaxis (Doctoral dissertation). The University of Mississippi, Mississippi (2024).

[13] Zhang C Song J Zheng Y Liu Y Chen Z. Nurses' knowledge and prescribing practices of HIV PrEP in the United States. J Assoc Nurses AIDS Care. (2023) 34:145–53. doi: 10.2139/ssrn.3566184

[14] Carter MR Aaron E Nassau T Brady KA. Knowledge, attitudes, and PrEP prescribing practices of health care providers in Philadelphia, PA. J Prim Care Community Health. (2019) 10:1–6. doi: 10.1177/2150132719878526 PMC6777052 31578913

[15] Pleuhs B Quinn KG Walsh JL Petroll AE John SA. Health care provider barriers to HIV pre-exposure prophylaxis in the United States: a systematic review. AIDS Patient Care STDS. (2020) 34:111–23. doi: 10.1089/apc.2019.0189 PMC7087402 32109141

[16] Antonini M Silva HCE Gerin L Cunha-Oliveira A Reis RK. Barriers to Pre-Exposure Prophylaxis (PrEP) use for HIV: an integrative review. Revista Brasileira De Enfermagem. (2023) 76:20210963. doi: 10.1590/0034-7167-2021-0963pt PMC10292616 37377313

[17] Mekonnen G Liknaw T Anley A Afenigus AD. Knowledge, attitudes, and associated factors towards HIV pre-exposure prophylaxis among health care providers in Ethiopia. Sci Rep. (2024) 14:6168. doi: 10.1038/s41598-024-56371-0 PMC10940609 38485990

[18] Habtie TE Kitaw TA Taye AB Adisu MA. Global insights into healthcare providers' knowledge, attitudes, practices, and concerns on pre-exposure prophylaxis: a systematic review and meta-analysis. Prev Med Rep. (2025) 60:103304. doi: 10.1016/j.pmedr.2025.103304 PMC12648478 41312424

[19] Feinberg J Keeshin S. Prevention and initial management of HIV infection. Annals of internal medicine. (2022) 175:ITC81–96. doi: 10.7326/AITC202206210 36534999

[20] Dlatu N Longo-Mbenza B Oladimeji KE Apalata T. Developing a model for integrating of tuberculosis, human immunodeficiency virus and primary healthcare services in Oliver Reginald (OR) Tambo District, Eastern Cape, South Africa. Int J of Environ Res and Public Health. (2023) 20:5977. doi: 10.3390/ijerph20115977 PMC10252508 37297581

[21] Hansoti B Mishra A Rao A Chimoyi L Redd AD Reynolds SJ . The geography of emergency department-based HIV testing in South Africa: Can patients link to care? eClinicalMedicine. (2021) 40:101091. doi: 10.1016/j.eclinm.2021.101091 PMC8548925 34746712

[22] Shamu S Khupakonke S Farirai T Slabbert S Chidarikire T Goloba G . Knowledge, attitudes and practices of young adults towards HIV prevention: an analysis of baseline data from a community-based HIV prevention intervention study in two high HIV burden districts, South Africa. BMC Public Health. (2020) 20:1249. doi: 10.1186/s12889-020-09356-3 PMC7433171 32807116

[23] Bailey VC Kleinhans AV Mokgatle MM. Knowledge and attitudes of HIV pre-exposure prophylaxis among nurses in South Africa. Afr J Prm Health Care & Fam Med. (2023) 15:4086. doi: 10.4102/phcfm.v15i1.4086 PMC10698804 37916728

[24] Akingbohungbe OO Yogeswaran P Adeleke OA. Knowledge, attitudes, and practices of primary care nurses regarding human immunodeficiency virus pre-exposure prophylaxis in the Mhlontlo Sub-District Municipality, Eastern Cape, South Africa. South African Family Practice. (2025) 67:6204. doi: 10.4102/SAFP.v67i1.6204 PMC12817032 41537575

[25] Eastern Cape Department of Health. District Health Plan OR Tambo District. Bhisho: ECDoH (2022).

[26] O.R Tambo District Municipality. Profile and analysis district development model. South Africa Department of Cooperative Governance and Traditional Affairs; OR Tambo District Municipality: Transkei District, South Africa (2020). Available online at: https://www.cogta.gov.za/ddm/wp-content/uploads/2020/11/ORTamnco-September-2020.pdf (Accessed June 12, 2025).

[27] Center for Disease Control and Prevention. Epinfo. (2019). Available online at: https://www.cdc.gov/epiinfo/index.html (Accessed April 14, 2025).

[28] Human Sciences Research Council. SABSSM VI. Provincial Dialogue. Eastern Cape media pack (2022). Available online at: https://hsrc.ac.za/sabssm-vi-provincial-dialogue-eastern-cape-%20media-478 pack (Accessed August 16, 2025).

[29] Ntimani JM. Mokoena-de Beer AG, Phetlhu DR. Exploring resource availability for nurses implementing HIV prevention guidelines in primary healthcare facilities. Health SA Gesondheid. (2025) 30:3084. doi: 10.4102/hsag.v30i0.3084 PMC12421553 40936642

[30] Ngwoke I Adedire AM Danladi NP Eze CC Dada OK Okesanya OJ. Barriers and facilitators of oral PrEP uptake against HIV', Current HIV/AIDS Reports. (2026) 23:14. doi: 10.1007/s11904-026-00783-2 PMC13035875 41910679

[31] Schmidt HM Schaefer R Radebe M Sued O Rodolph M Ford N. Baggaley R. Scaling up access to HIV pre-exposure prophylaxis (PrEP): should nurses do the job? The Lancet HIV. (2022) 9:e363–6. doi: 10.1016/S2352-3018(22)00006-6 PMC9046094 35358418

[32] Henderson M Schmidt H-MA Chitembo L Peralta H Alaama AS Johnson C . The future of pre-exposure prophylaxis (PrEP) for HIV prevention: a global qualitative consultation on provider perspectives on new products and differentiated service delivery. AIDS Behavior. (2023) 27:3755–66. doi: 10.1007/s10461-023-04093-1 PMC10589125 37351685

[33] Gengiah TN Naidoo V Dlamini N Khan M Khanyile SS Nxumalo SP . Willingness to switch to injectable cabotegravir among oral PrEP users in Durban, South Africa. Health SA Gesondheid. (2025) a3120. doi: 10.4102/HSAG.v30i0.3120 PMC12587083 41200005

[34] World Health Organization. Update on HIV Prevention Guidelines: Expanding PrEP Choices. Geneva: WHO (2024).

